# Risk Factors for Life‐Threatening Asthma Attacks and Asthma‐Related Mortality in Children—A Systematic Review

**DOI:** 10.1002/ppul.71255

**Published:** 2025-08-19

**Authors:** Aleksandra Gawlik‐Lipinski, Sarah Hassen, Manisha Ramphul, Erol A. Gaillard, Jenni K. Quint, Clare L. Gillies, David Lo

**Affiliations:** ^1^ University of Leicester Leicester UK; ^2^ University Hospitals of Leicester NHS Trust Leicester UK; ^3^ Imperial College London London UK

**Keywords:** asthma death, fatal asthma, ICU admission, near fatal asthma attack, pediatric

## Abstract

**Background:**

Awareness of factors associated with life‐threatening asthma attacks and asthma mortality is essential for risk prediction in asthma care. There is limited evidence to inform risk factors in children and young people (CYP). The aim of this systematic review is to synthesize existing data to report risk factors for life‐threatening asthma attacks and asthma deaths in CYP.

**Methods:**

This review followed PRISMA‐reporting‐guidelines and was registered on PROSPERO (CRD42023494203). We included observational studies of CYP aged 0−18 years with asthma, published in English, from 2000 to the present, exploring variables influencing asthma mortality and life‐threatening attacks (defined as intensive care [ICU] admissions). CINAHL, MEDLINE, Central Cochrane Library, and Scopus databases were searched. Reference lists of eligible studies were screened. The MEDLINE search strategy was adapted across the databases. Results were synthesized narratively.

**Results:**

Six studies met our inclusion criteria (five retrospective cohort, one case‐control study). All were conducted in high‐income countries. In descending order of strength of association, factors found to be associated with ICU admissions were: high salbutamol use, previous hospitalization, low socioeconomic status, allergies, Black ethnicity, prescription of asthma medications, history of pneumonia, rural location, adolescence, paternal asthma, and co‐existing comorbidity.

For the outcome of mortality, one study reported a sevenfold risk of asthma death in children of Black ethnicity compared with White.

The included studies had low quality of evidence by the GRADE assessment.

**Conclusion:**

The small number and high heterogeneity between available studies, identifies a need for robust epidemiological studies and better risk prediction for near‐fatal and fatal attacks in CYP‐asthma.

## 1 Introduction

Asthma is an inflammatory disease of the airways, presenting with wheeze, shortness of breath, chest tightness, and cough, and characterized by variable and reversible airflow obstruction [1, 2]. It is estimated that 262 million people have been diagnosed with asthma globally [3], and 8 million people in the United Kingdom [4].

A sudden symptom deterioration, known as an “asthma attack,” may be triggered by respiratory infections, aeroallergens, physical activity, or weather changes [1, 2, 5]. Even with prompt management, attacks can be life‐threatening or fatal [1]. Current guidelines [1, 2, 5] emphasize the need to assess the risk of future asthma attacks in addition to current symptom control at every review, highlighting that symptom control and attack risk may be discordant.

In school‐aged children, a history of a previous asthma attack is the strongest predictor for further attacks [6]. This is why an early asthma review is recommended following every attack [2, 5].

Despite these recommendations, around 1000 people die of fatal asthma attacks every day globally [7], 1200 people annually in the United Kingdom [8], and around 3235 people annually in the United States of America [9].

In the United Kingdom, which has one of the highest incidences of asthma deaths in children and young people (CYP) amongst high‐income countries [10], a detailed review of asthma‐related deaths was commissioned in 2014. The National Review of Asthma Deaths (NRAD) [8] scrutinized the medical records of 195 people (including 28 children) who died from asthma between 2012 and 2013. The review identified potentially avoidable factors in 67% of asthma deaths, including lack of asthma reviews, over‐reliance on asthma reliever inhalers (short‐acting beta‐2 agonists, SABA), and lack of written asthma action plans [8]. A recent report from the National Child Mortality Database [11] reported 54 asthma deaths over a 4‐year period in the United Kingdom. High deprivation levels, air pollution, high use of SABA, previous hospitalization or emergency department visits, and prematurity were some of the risk factors identified in the report [11]. As these reviews only included people who died, we do not know whether these factors are unique to that population or ubiquitous amongst people with frequent asthma attacks.

In addition to assessing patients for their risk of asthma attacks, it is clearly important to identify risk factors for asthma deaths and life‐threatening attacks. Therefore, this review aims to synthesize and summarize evidence to inform risk factors and predictors for life‐threatening and fatal asthma attacks in CYP from studies published from 2000 onwards.

## 2 Methods

This systematic review followed the Preferred Reporting Items for Systematic Reviews and Meta‐Analyses (PRISMA) reporting guidelines [12] and was registered on PROSPERO (#CRD42023494203).

### 2.1 Data Sources and Search Strategy

Searches were conducted on January 18, 2024, and updated on September 5, 2024, using the CINAHL, MEDLINE, Central Cochrane Library, and Scopus databases.

The MEDLINE search strategy was adapted for each search. Medical Subject Headings (MeSH) terms (exploded) and free text terms corresponding to the population: “asthma*” and “child,*” exposure of “risk,*” and outcomes of “mortality” and “life‐threatening” were explored. For improved sensitivity, a truncation asterisk (*) was applied. The search strategies (S1) were consulted by a specialist librarian.

### 2.2 Eligibility Criteria and Selection Process

We included studies meeting the following criteria: (1) Observational studies published in English since 2000; (2) concerning CYP aged 0−18 with asthma; (3) reporting any physiologically plausible variable(s) associated with the outcomes of interest:

1. Asthma‐related mortality or
2. Life‐threatening asthma (defined as an intensive care unit [ICU] or high dependency care unit [HDU] admission with asthma).

Case series, editorials, review articles, and conference proceedings were excluded.

### 2.3 Literature Screening

Search results were imported onto the Rayyan systematic review platform [13]. After screening and manually removing duplicates, title and abstract screening was conducted to remove ineligible studies. Full‐texts of the remaining references were retrieved for screening. Four reviewers (A.G.L., S.H., M.R., D.L.) were involved. All stages were blinded and conducted by at least two reviewers independently following the pre‐defined inclusion/exclusion criteria (Table 1). Conflicts were resolved by discussion between reviewers.

**Table 1:** Inclusion and exclusion criteria for studies.

Inclusion	Exclusion
Study design: Observational studies, for example,	Case series
Case‐control	RCTs
Cross‐sectional	Editorials, reviews
Cohort (retrospective and prospective)	Conference proceedings
Population	Population
Children aged 0−18	Adults (18+)
	Children with another respiratory comorbidity, such as cystic fibrosis, primary ciliary
Children diagnosed with asthma	Dyskinesia and so forth.
Exposures	Exposures
Studies reporting variables—risk factors or predictors for fatal or near‐fatal asthma	Studies not reporting associated variables/risk factors
Outcome	Outcome
Asthma mortality (fatal asthma)	Not reporting outcomes of interest
Life‐threatening asthma attack (defined as ICU or HDU admission)	
Language	
Published in English	Studies reporting predominantly on data from before 2000
Using predominantly data obtained in 2000 or later	
Published after 2000	

### 2.4 Data Extraction

AGL extracted data into a pre‐designed data collection form, which was checked/verified by D.L. Respective authors were contacted for any missing data, and if any assumptions were made, it is clearly stated. Data extracted included:

1. Study characteristics: Author, publication year, country, study design/type, population, funding, and conflicts of interest.
2. Risk factors, relative risk estimates (if reported), or number of events in each comparator group for outcomes.
3. Outcomes of interest (asthma‐related mortality and asthma‐related ICU/HDU hospitalization).

### 2.5 Risk of Bias and Quality Assessment

Risk of bias was assessed using the ROBINS‐E tool for observational studies [14], while the quality and strength of the evidence using the GRADE tool, specifically the guidelines [15] on applying GRADE in the “absence of a single estimate of effect.”

### 2.6 Effect Measures

We included studies reporting (but not limited to) odds ratios (OR), risk ratios (RR), and hazard ratios (HR) for the outcomes of interest. For studies reporting adjusted and non‐adjusted outcome measures, we included the adjusted values.

### 2.7 Synthesis Methods

The data were aggregated and synthesized thematically using narrative synthesis [16]. Quantitative synthesis was not feasible as the studies utilized different reporting measures and assessed the risks using different methods which precluded meta‐analysis.

## 3 Results

**Figure 1:**
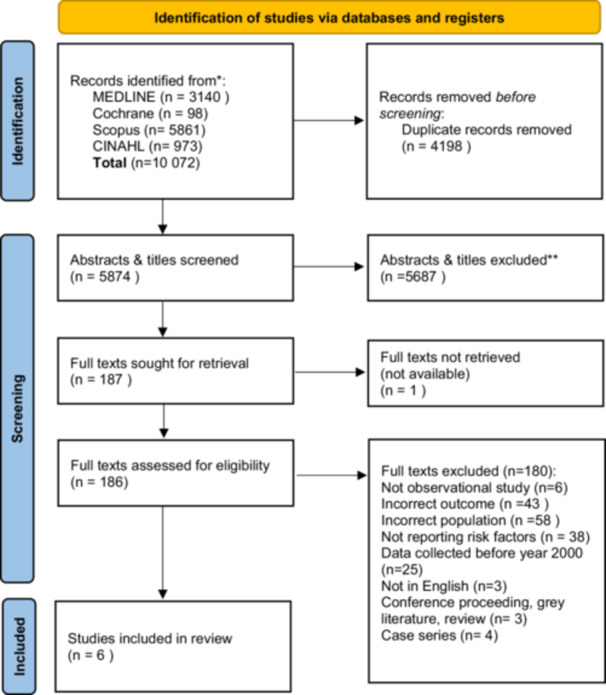
PRISMA flowchart for the identification of studies via databases and registers, including combined searches run on January 18, 2024, and September 5, 2024. [Color figure can be viewed at wileyonlinelibrary.com]

Our searches identified 10,072 studies. After duplicate removal, 5874 titles and abstracts were screened. Of the 186 full‐texts assessed for eligibility, only six studies were eligible for inclusion. Reasons for exclusion are depicted in Figure 1.

### 3.1 Study Characteristics

#### 3.1.1 Design, Participants, and Setting

All six included studies were from high‐income countries (two from the USA [17, 18], and the remaining four from Korea, Canada, the Netherlands, and the UK, respectively [19, 20, 21, 22]). They were conducted between 1994 and 2019 and published between 2012 and 2024. One was a retrospective case‐control study [21], and five were retrospective cohort studies [17, 18, 19, 20, 22].

Participants with asthma were recruited from outpatient clinics or the general population. Cohort sizes ranged from 579 to 4,500,000, and the ages of the participants varied from 0‐to‐18 across the studies.

#### 3.1.2 Outcomes

Only one study [17] reported risk factors for asthma mortality, while the remaining five reported on risk factors for life‐threatening asthma. Study characteristics are summarized in Table 2.

**Table 2:** Characteristics of studies included in the systematic review.

#	Author & year	Location	Study design	Population & setting	Sample size	Age	Years	Risk factors assessed
1	Akinbami et al. (2014)	USA	Retrospective cohort study	Children with asthma (asthma coded based on questionnaire response)	4.5 min (asthma prevalence 2010)	0−17	2001−2010	Black ethnicity
2	Grunwell et al. (2018)	USA	Retrospective cohort study	Children with physician‐diagnosed asthma, quaternary children′s care center (outpatient)	579	6−18	2004−2015	Black ethnicity, other ethnicity, low socioeconomic status, pneumonia, severe asthma/high ICS use, father's asthma, hospitalized in 12 months before survey
3	Lee et al. (2023)	Korea	Retrospective cohort study	Children and adolescents with physician‐diagnosed asthma	1,537,066	2−18	2013−2019	Low socioeconomic status
4	Radhakrishnan et al. (2018)	Canada	Retrospective cohort study	10 separate consecutive cohorts of children born between April 1, 1996 and March 31, 2006 in Ontario with physician‐diagnosed asthma	254,964 children with asthma	0−8	1996−2013	Low socioeconomic status, year of birth, age at diagnosis, gender (male vs. female), comorbidity, treated by pediatrician versus non‐pediatrician, rural versus urban, instability quantile, dependency quantile, ethnic concentration quantile, deprivation quantile
5	van den Bosch et al. (2012)	Netherlands	Case‐control study	Children with physician‐diagnosed asthma seen in one of four outpatient clinics	230 (66 cases, 164 controls)	0.6−16.5	1994–2006	Sanitized home, allergies, passive smoking, earlier hospitalization for asthma (non‐PICU)
6	Simms‐Williams and Nagakumar (2024)	UK	Retrospective cohort study	Children aged 5+ with physician‐diagnosed asthma registered with a general practice submitting data to Clinical Practice Research Datalink	205,916	5−17	2017− 2019	Black ethnicity, mixed ethnicity, low socioeconomic status, gender (female vs. male), SABA prescriptions, OCS, ICS, LTRA

Abbreviations: ICS, inhaled corticosteroid; LTRA, leukotriene receptor antagonist; OCS, oral corticosteroids; SABA, short‐acting beta‐2 agonist.

#### 3.1.3 Risk Factors

Included studies explored a wide range of potential risk factors, including age, sex, ethnicity, socioeconomic status (SES), comorbidities, family history, hospitalization, medication, environmental factors, and treating clinician credentials.

### 3.2 Risk of Bias and Quality Assessment

Only one study, Radhakrishnan et al. [20] did not raise concerns about risk of bias. The remaining studies had variable risks of bias primarily related to selection bias [18, 19, 22] and missing data [17, 19, 22]. Akinbami et al. had a high risk of bias due to confounding [17], and van den Bosch et al. had a very high risk of bias due to inappropriate measurement of exposures: smoking and sanitized homes. Passive smoking was recorded based on proxy reporting, and the house was deemed sanitized if it was visited by an asthma nurse at any time before the admission, and advice was given on how to sanitize it. There is a lack of information on whether the implementation of the advice was checked [21]. Risk of bias assessment is depicted in Supporting Information S2: Tables S1 and S2.

Based on the GRADE assessment (Supporting Information S2: Table S3 and S4) to inform our outcomes of interest, the overall quality of evidence was judged to be low, with serious risks of bias and a low level of precision.

### 3.3 Risk Factors for Asthma Death

Only Akinbami et al. [17] reported risk factors for pediatric asthma mortality. The authors reported a 7.1‐fold risk of asthma deaths in children of Black compared with White ethnicity (population‐based ratio PBR 7.1, 95% CI 5.2−9.7). Asthma death as an outcome was calculated “using the entire population of children in an ethnic group as the denominator” [17].

### 3.4 Risk Factors for Life‐Threatening Asthma Attack

Five studies [18, 19, 20, 21, 22] reported factors associated with ICU admissions as a surrogate for life‐threatening asthma attacks.

#### 3.4.1 Previous Hospitalization

Grunwell et al. [18] and van den Bosch et al. [21] both reported a positive association between previous hospitalization and odds of ICU admission (OR 8.19, 95% CI 4.83−13.89, *p* < 0.001; OR 5.40, 95% CI 1.34−21.45, *p* = 0.02, respectively). It should be noted that “previous hospitalization” was defined differently between studies. Grunwell et al. [18] reported hospitalizations in the last 12 months, whereas van den Bosch et al. [21] reported any previous non‐ICU admission.

#### 3.4.2 Socioeconomic Background

Four studies [18, 19, 20, 22] reported a positive association between low SES and risk of ICU admission (Grunwell: OR 1.28, 95% CI 1.02−1.61, *p* = 0.037; Lee: aHR 7.12, 95% CI 3.72−13.62, *p* not reported; Radhakrishnan: OR 1.16, 95% CI 1.07−1.27, *p* < 0.005; Simms‐Williams: IRR 1.98, 95% CI 1.03−3.79, *p* = 0.041 for ages 5−11). However, the definitions and units of measurement for SES differed between studies, introducing a high level of heterogeneity.

Grunwell reported the association between ICU admissions and the proportion of people living below the poverty line (not defined) in each child's zip (postal) code [18]. Lee et al. divided their cohort into five SES groups based on their level of health insurance cost. From SES 0 (those on medical aid) to SES 4 (those with the highest medical insurance premiums).

Radhakrishnan [20] reported SES using the Ontario Marginalization Index—an area‐based measure of SES based on four dimensions (household types, material resources, age and labor force, and racialised and newcomer populations), and Simms‐Williams and Nagakumar [22] used UK index of multiple deprivation (IMD) quintiles (1 is the least and 5 the most deprived).

#### 3.4.3 Allergies

Two studies [21, 22] explored allergies as a potential risk factor for ICU admissions.

The van den Bosch study reported a positive association between the presence of allergy (defined as evidence of senitization to several antigens including food, dust mites, animals, based on blood or skin‐prick tests) and increased odds of ICU admission with acute asthma in children (OR 5.2, 95% CI 1.14–23.42, *p* = 0.03). Conversely, Simms‐Williams and Nagakumar observed no association between allergies (based on documentation in clinical records) with ICU admissions in either the 5−11 (IRR 1.16, 95% CI 0.74−1.83, *p* = 0.52) or the 12−17 year age‐group (IRR 1.51, 95% CI 0.89−2.54, *p* = 0.124).

#### 3.4.4 Ethnicity

Two studies [18, 22] explored the association between ethnicity and CYP asthma ICU admissions. Both studies observed an increased risk of ICU admission amongst children of Black compared with White ethnicity.

Grunwell et al. [18] categorized their cohort into three ethnic groups comprising mostly children from a Black (58%) ethnic background, followed by White (30%) and other (12%). Compared with children of White ethnicity, children of Black ethnic background had twofold higher odds for an ICU admission for acute asthma (OR of 2.01, 95% CI 1.05−3.84, *p* = 0.034).

Simms‐Williams and Nagakumar [22] categorized their cohort into groups of children from Black, mixed, Asian, White (reference category), and other ethnic backgrounds. Children of Black ethnicity had higher incidence rates for ICU admissions compared with white children in both those aged 5−11 years (IRR 4.07, 95% CI 2.35−7.05, *p* < 0.001) and aged 12−17 years (IRR 3.51, 95% CI 1.62−7.59, *p* = 0.001). Children aged 5−11 from a mixed ethnic background also had a higher incidence rate for ICU admission (IRR 2.49, 95% CI 1.23−5.05, *p* = 0.011) but not older children aged 12−17 [22].

#### 3.4.5 Sex

The association between sex and ICU admissions was reported in two studies [20, 22].

Radhakrishnan et al. observed no difference in the odds of ICU admissions in male compared with female children (OR 0.93, 95% CI 0.76−1.15, *p* = 0.5) aged 0−8 years. Simms‐Williams and Nagakumar reported an increased risk of ICU admissions in older female children aged 12−17 years (IRR 1.54, 95% CI 1−2.36, *p* = 0.05) but no difference between sexes in younger children aged 5−11 years (IRR 1.20, 95% CI 0.85−1.7, *p* = 0.306).

#### 3.4.6 Age

Two studies [18, 20] explored the association between age and ICU admission.

Grunwell et al. [18] reported a higher risk of ICU admission in children over 12 years compared with children 12 years and under (OR 2.31, 95% CI 1.39−3.86, *p* = 0.001). Radhakrishnan et al. [20] only included children aged 0−8 in their cohort and reported an 8% reduction in the odds of an ICU admission with each 1‐year increase in age at diagnosis (OR 0.92, 95% CI 0.87−0.97, *p* < 0.005).

#### 3.4.7 Comorbidities

Three studies [18, 20, 22] explored the association between comorbidities and ICU admission. Pneumonia (OR 2.56, 95% CI 1.52−4.29, *p* < 0.001) [18] and presence of any comorbidity defined as prematurity, congenital heart disease, or any chronic lung disease (OR 1.87, 95% CI 1.44−2.42, *p* < 0.005) [20] were reported as having a positive association with ICU admissions. In the Simms‐Williams and Nagakumar study [22], multiple variables were explored, including body mass index (BMI), atopic conditions, gastro‐esophageal reflux disorder (GORD), chronic rhinosinusitis, and mental health conditions, yet none of them were found to be associated with ICU admissions.

#### 3.4.8 Medication

The association between prescription medicines and admission to the ICU was considered in two studies [18, 22].

Grunwell et al. [18] reported increased odds of ICU admission in children prescribed high doses of inhaled corticosteroids (ICS, defined as inhaled fluticasone equivalent > 800 mcg if 12 years or > 400 mcg if under 12 years old) (OR 2.76, 95% CI 1.62−4.70, *p* = 0.001).

In the Simms‐Williams and Nagakumar [22] study, there was an increased risk of ICU admission in children aged 12−17 prescribed any dose of ICS (IRR 3.95, 95% CI 1.40−11.17, *p* = 0.009) in the previous year. Prescription of oral corticosteroid (OCS) at any dose and duration within 1 year before the index date was also associated with increased incidence rates for ICU admission in children aged 5−11 (IRR 1.98, 95% CI 1.34−2.92, *p* = 0.001) and 12−17 (IRR 4.06, 95% CI 2.56−6.45, *p* < 0.001). Furthermore, there was a positive association between the use of leukotriene receptor antagonists (LTRA) and ICU admissions in both age groups (IRR 2.86, 95% CI 1.93−4.24, *p* < 0.001 in the 5−11; IRR 2.77, 95% CI 1.71−4.49, *p* < 0.001 in the 12−17 age group).

Additionally, Simms‐Williams and Nagakumar examined the association between the number of SABA prescriptions and ICU admissions. They observed an increased risk in children 5−11 years of age receiving 5−6 or more than 7 SABA inhalers/year (IRR 5.21, 95% CI 2.26−12.01, *p* < 0.001; IRR 6.97, 95% CI 2.91−16.68, *p* < 0.001, respectively). In 12–17‐year‐olds, the association was significant only in those receiving seven or more SABA inhalers/year (IRR 8.44, 95% CI 2.49−28.56, *p* = 0.001).

#### 3.4.9 Environmental Factors (Incl. Geographical Location)

Two studies [20, 22] examined environmental factors potentially impacting ICU admissions. Radhakrishnan et al. [20] reported increased odds of ICU admission in children living rurally, defined as areas with less than 10,000 inhabitants (OR 2.42, 95% CI 1.80−3.25, *p* < 0.005), while Simms‐Williams and Nagakumar [22] examined whether smoking has an impact on ICU admissions in the age group of 12−17, but observed no association.

#### 3.4.10 Additional Reported Factors

Other factors explored included treating clinician, and paternal asthma. Grunwell et al. [18] reported a 2.15‐fold risk of ICU admission in children having a father with asthma (OR 2.15, 95% CI 1.23−3.76, *p* = 0.007). As reported by Radhakrishnan et al. [20] being treated by a pediatrician during outpatient visits in the 2 years preceding the admission lowered the risk of ICU admission compared to children treated by a non‐pediatrician (OR 0.74, 95% CI 0.58−0.94, *p* = 0.007). See S3 results tables.

## 4 Discussion

### 4.1 Principal Findings

This systematic review aimed to identify risk factors for life‐threatening asthma attacks and asthma death in CYP. The greatest risk factor for ICU admission (eightfold higher risk) was high‐SABA use ( ≥ 7 inhalers/annum) [22] and hospitalization for asthma within the previous 12 months [18]. Furthermore, low SES (living on medical aid) increased the risk sevenfold [19], while allergies (to food, dust mites, animals) were associated with fivefold higher risk [21].

Children of Black ethnic background were at fourfold higher risk of ICU admission compared to those of White background [22]. Black CYP were found to have a sevenfold greater risk of dying from asthma compared with CYP from a White ethnic background. This data is, however, derived from a single study, and did not include any other ethnic groups.

Other important risk factors identified for life‐threatening asthma (in descending order of risk) included: prescription of OCS within the past year [22], high dose of ICS [18, 22], or LTRA [22]; history of pneumonia ever [18], rural location [20], adolescent age, paternal asthma [18], and co‐existing comorbidity (prematurity, congenital heart disease, chronic lung disease, history of pneumonia) [18, 20]. It should be noted that the observed association between OCS, ICS, and LTRA prescriptions with ICU admissions is likely indicative of prescription patterns in children with more severe or uncontrolled asthma.

### 4.2 Comparison With Other Studies

The findings of our review echo factors identified in previous UK national reports on asthma deaths [8, 11]. Although none of the studies identified were mechanistic, the associations observed are physiologically plausible.

Previous hospitalization and/or high SABA use likely reflect poor asthma control. High SABA use is associated with a greater risk of asthma death [23] and over‐reliance on reliever medications is often associated with underuse of regular ICS [24]. These factors have also been reported in a previous systematic review [25] and in multiple individual studies [26, 27, 28]. However, these studies were not included in our review due to their cohorts being derived from hospital inpatients rather than the general population. This introduces a risk of selection bias and may not reflect the general population of children with asthma that we were interested in.

Deprivation is associated with worse outcomes in multiple chronic disease areas [29, 30, 31, 32, 33, 34]. Hypothesized reasons for this are multifactorial, but include barriers to accessing healthcare (such as lack of transportation), living in areas of poorer air quality, and risk‐taking behaviors within the household (such as smoking).

Non‐White ethnicity has also been implicated in poor asthma outcomes in previous studies [29, 35, 36]. The reasons for this are less clear, and may be confounded by socioeconomic factors, cultural beliefs, health literacy, and language barriers.

Female sex as a risk factor for asthma attacks has been reported in previous studies [37, 38], and is believed to be associated with hormonal changes occurring during puberty and the impact of hormones on airway inflammation. Simms‐Williams and Nagakumar reported a higher risk of ICU admissions in females, contrary to Radhakrishnan et al. [20] who reported no difference between sexes. The latter study, however, only included pre‐pubertal children in whom ovarian hormone levels are low.

Higher risk of ICU admissions in adolescence was reported in one of the studies [20]. This may be related to hormonal changes in puberty, but also risk‐taking behaviors such as smoking or vaping. Furthermore, it might be speculated that adolescents, with increasing autonomy, may no longer be supervised to take their preventer medications regularly, resulting in poorer asthma control.

Atopy and allergy as comorbidities have been explored in multiple studies. One study included in our review reported an over fivefold higher risk of ICU admission in children with allergies (food, dust mites, animals) [21]. This study, however, had a very high risk of bias. Allergies, particularly indoor allergens such as animal dander and house dust mites, were reported as high‐risk factors by McDowell et al. [39]. The latter study, however, examined the risk factors in children who were inpatients in the hospital and were more acutely ill at their baseline than our population of interest.

A previous systematic review by Alvarez et al. [25] found no significant association between the presence of atopy and asthma‐related ICU admissions.

The findings between our study and that reported by Alvarez et al. may differ due to the source data being retrieved from different time periods (our study predominantly with data from after 2000 and Alvarez from before 2000), as well as the studies included in Alvarez's review not being limited to the pediatric population.

Extremes of BMI were found to be associated with ICU admissions in two of our included studies [40, 41], but not in the study by Simms‐Williams and Nagakumar [22]. Mechanisms for this observed association are likely multifactorial, possibly related to common causal pathways for both asthma attacks and obesity or malnutrition, including deprivation, access to healthcare, exercise, and food choices [42]. Obesity is additionally associated with increased levels of systemic inflammation [40], which may increase asthma exacerbation risk.

Interestingly, active smoking, albeit reported only in one study [22], was not found to be a major contributing factor to life‐threatening asthma in our systematic review, contrary to a previous report [8]. This might be related to the limitations of the routine primary healthcare database used in the one observational study included in our review reporting on smoking risk [22]. Smoking might not have been correctly or incompletely recorded in patients' electronic records (particularly exposure to second‐hand tobacco smoke in children). Second‐hand smoking, reported as a risk factor in the literature [8] was not explored by any of the studies.

None of the included studies reported on the impact of air pollution on adverse asthma outcomes. High air pollution, the presence of particulate matter (PM) 2.5 and ozone, was reported as a risk factor for asthma deaths by Mirabelli et al. [43]. A recent systematic review of observational studies focusing on ambient air pollution by Varghese et al. [44] reported a possible association between PM2.5 and ozone and near‐fatal asthma attacks in children. Neither of the above studies met our inclusion criteria, with Mirabelli et al. [43] being a case series (80 coroner report reviews), and Varghese et al. [44] being a systematic review of four observational studies within an ICU setting.

Finally, only one statistically significant protective factor was identified—being treated by a pediatrician [20]. It could be hypothesized that referral to a secondary or tertiary care setting benefits the patient with more regular follow‐ups, multidisciplinary input, facilitated self‐management, better availability of objective testing, therefore allowing for earlier diagnosis and treatment, reducing the risk of severe attacks.

### 4.3 Strengths

This review followed a protocol and PRISMA reporting guidelines and utilized ROBINS‐E and GRADE assessment for study quality. Experts were consulted on search strategies (specialized librarian) and analysis methods (medical statistician). To reduce the risk of selection bias, the population of interest were any CYP with asthma, to reflect the risks within the asthma population, rather than the risk of poor outcomes only in those already hospitalized.

### 4.4 Limitations

While our review followed a robust methodology, we recognize it has limitations. First, we included only studies available in English, which may have missed eligible studies from non‐English populations. Second, very few studies met the pre‐set inclusion criteria, and due to the heterogeneity of the included studies, meta‐analysis was not possible. The included studies were observational, and the GRADE assessment [15] revealed low‐quality evidence. Furthermore, the definition of asthma varied across the studies (e.g., asthma reported by proxy, based on ICD codes, etc.), and only one study [20] did not raise concerns about the risk of bias. All studies had a level of imprecision, and due to their heterogeneity, no pooled quantitative analysis was plausible. Also, the studies were conducted in high‐income countries; hence, the results might not be applicable in lower‐income countries, where different risk factors might be more prominent. Only one study reported on mortality, which only explored ethnicity as a risk factor and did not account for potential confounders. None of the studies meeting the search criteria analyzed the correlation between previously collected physiological data, air pollution, medication adherence, and near‐fatal and fatal asthma.

### 4.5 Conclusions and Implications for Practice

Our detailed review of the literature identified very few studies exploring risk factors for life‐threatening asthma attacks in children, and only one exploring asthma deaths. All these were conducted in high‐income countries, and most had a risk of bias and imprecision. Our findings highlight the scarcity of evidence to inform risk prediction in pediatric asthma, and the need for well‐designed epidemiological studies to identify factors for fatal and near‐fatal asthma in CYP.

Despite the limitations of the included studies, we identified several physiologically plausible risk factors for life‐threatening asthma attacks, including excess SABA usage and previous attacks which are consistent with findings from previous reviews. The presence of these factors should prompt an urgent clinical review with an asthma‐trained professional to optimize management and control. The current individual risk factors identified are nonspecific, and more detailed risk prediction is needed for clinicians to aid clinical decision making and recognition of individuals at a high risk of life‐threatening attacks and asthma deaths.

## Author Contributions

**Aleksandra Gawlik‐Lipinski:** conceptualization, methodology, records screening, data extraction, data curation, analysis and synthesis, writing – original draft. **Sarah Hassen:** records screening, writing – review and editing. **Manisha Ramphul:** records screening, writing – review and editing. **Erol Gaillard:** writing – review and editing. **Jenni Quint:** supervision, methodology, analysis‐review, writing – review and editing. **Clare Gillies:** supervision, methodology, analysis, reviewing, writing – review and editing. **David Lo:** conceptualization, supervision, methodology, records screening, data extraction, review, analysis, writing – review and editing.

## Ethics Statement

The authors have nothing to report.

## Conflicts of Interest

Erol Gaillard: Investigator‐led research grants from Gilead Sciences, Chiesi Limited, and Propeller Health. Research collaboration with AstraZeneca, Helicon Health, and Adherium (NZ) Limited. Speaker fees, Circassia Group, and Sanofi. Aleksandra Gawlik‐Lipinski: Speaker fees and conference honoraria from AstraZeneca and Chiesi. The other authors declare no conflicts of interest.

## Supporting information

Supplement 1 ‐ Search strategies.

Supplement 2 ‐ Risk of bias GRADE assessment.

Supplement 3 ‐ Risk factors for ICU admission‐tabular presentation.

## Data Availability

The data that supports the findings of this study are available in the supplementary material of this article.
