# Supplementary material for: Risk Factors for Life‐Threatening Asthma Attacks and Asthma‐Related Mortality in Children—A Systematic Review
Source: Pediatr Pulmonol. 2025 Aug 19;60(8):e71255. doi: 10.1002/ppul.71255 (PMC12391745; doi:10.1002/ppul.71255)
Supplement: Supplementary file 1 — Supplement 1 ‐ Search strategies. [file PPUL-60-0-s001.docx]

# Supplement 1

Search terms and strategy

| Risk factors for asthma-related mortality and life-threatening attacks in children and adults | | | | |
| --- | --- | --- | --- | --- |
| Population | AND | Intervention/exposure | AND | Outcome |
| Children with asthma | AND | Risk | AND | Mortality  Life-threatening |
| Asthma*    AND    Child  OR  Children  OR  Pediatric  OR  Paediatric  OR  Infant*  OR  Toddler*  OR  Teenager*  OR  Baby  OR  Babies  OR  Adolescent*  OR  Preschooler* |  | Risk*  OR  Predictor*  OR  Possibilit*  OR  Probability*  OR  Likelihood  OR  Chance*  OR  Rate  OR  Rates  OR  Incidence |  | Mortality  OR  Death*  OR  Dying  OR  Life-Threatening  OR  Critical Care*  OR  Intensive Care*  OR  Intensive Treatment* |

## MEDLINE Search strategy

Ovid MEDLINE(R) ALL <1946 to January 17, 2024>

1 (Risk* or predictor* or possibilit* or probability* or likelihood or chance* or rate or rates or incidence).mp. [mp=title, book title, abstract, original title, name of substance word, subject heading word, floating sub-heading word, keyword heading word, organism supplementary concept word, protocol supplementary concept word, rare disease supplementary concept word, unique identifier, synonyms, population supplementary concept word, anatomy supplementary concept word] 7772323

2 exp Risk/ or exp Risk Factors/ 1399043

3 exp Probability/ 1620507

4 exp Incidence/ 304219

5 1 or 2 or 3 or 4 7874068

6 (mortalit* or fatalit* or death* or dying or life-threatening or life threatening or intensive care* or critical care* or intensive treatment*).mp. [mp=title, book title, abstract, original title, name of substance word, subject heading word, floating sub-heading word, keyword heading word, organism supplementary concept word, protocol supplementary concept word, rare disease supplementary concept word, unique identifier, synonyms, population supplementary concept word, anatomy supplementary concept word] 2565116

7 exp Mortality/ 425612

8 exp Death/ 166665

9 exp Critical Care/ 67607

10 6 or 7 or 8 or 9 2721522

11 exp Asthma/ or asthma*.mp. 206473

12 (child or children or pediatric* or paediatric* or adolescent* or toddler* or teenager* or preschooler* or baby or babies).mp. [mp=title, book title, abstract, original title, name of substance word, subject heading word, floating sub-heading word, keyword heading word, organism supplementary concept word, protocol supplementary concept word, rare disease supplementary concept word, unique identifier, synonyms, population supplementary concept word, anatomy supplementary concept word] 4055517

13 exp Child/ or exp Child, Preschool/ 2182860

14 exp Adolescent/ 2232364

15 exp Pediatrics/ 63225

16 infant.mp. or exp Infant/ 1330570

17 12 or 13 or 14 or 15 or 16 4555508

18 5 and 10 and 11 and 17 3317

19 limit 18 to english language 3075

## Scopus search strategy

"risk*" OR "predictor*" OR "possibilit*" OR "probability*" OR "likelihood" OR "chance*" OR "rate" OR "rates" OR "incidence" )

AND

 "mortalit*" OR "fatalit*" OR "death*" OR "dying" OR "life-threatening" OR "life threatening" OR "intensive care*" OR "critical care*" OR "intensive treatment*" )

AND

 asthma*

AND "child" OR "children" OR "pediatric*" OR "paediatric*" OR "adolescent*" OR "toddler*" OR "teenager*" OR "preschooler*" OR "baby" OR "babies" OR "infant*" OR "adolescen*"

Limit applied: English language

## CINAHL search strategy

( Risk* or predictor* or possibilit* or probability* or likelihood or chance* or rate or rates or incidence ) AND ( mortalit* or fatalit* or death* or dying or life-threatening or life threatening or intensive care* or critical care* or intensive treatment* ) AND asthma AND ( child or children or pediatric* or paediatric* or adolescent* or toddler* or teenager* or preschooler* or baby or babies or infant* or adolescen* )

Limit applied: English

## Cochrane search strategy

ID Search

#1 asthma*

#2 MeSH descriptor: [Asthma] explode all trees

#3 MeSH descriptor: [Mortality] explode all trees

#4 MeSH descriptor: [Death] explode all trees

#5 MeSH descriptor: [Critical Care] explode all trees

#6 dying or life-threatening or life threatening or intensive care or intensive treatment or mortalit* or death* or critical care or fatalit*

#7 child* or p*diatric* or adolesc* or preschooler* or infant* or baby or babies or toddler* or teenager* or teen*

#8 MeSH descriptor: [Child] explode all trees

#9 MeSH descriptor: [Pediatrics] explode all trees

#10 MeSH descriptor: [Infant] explode all trees

#11 MeSH descriptor: [Adolescent] explode all trees

#12 MeSH descriptor: [Risk] explode all trees

#13 MeSH descriptor: [Incidence] explode all trees

#14 Risk* or predictor* or possibilit* or probability* or likelihood or chance* or rate or rates or incidence

#15 MeSH descriptor: [Probability] explode all trees

#16 (#1 or #2) and (#3 or #4 or #5 or #6) and (#7 or #8 or #9 or #10 or #11) and (#12 or #13 #14 or #15)
