# Supplementary material for: Risk Factors for Life‐Threatening Asthma Attacks and Asthma‐Related Mortality in Children—A Systematic Review
Source: Pediatr Pulmonol. 2025 Aug 19;60(8):e71255. doi: 10.1002/ppul.71255 (PMC12391745; doi:10.1002/ppul.71255)
Supplement: Supplementary file 2 — Supplement 2 ‐ Risk of bias GRADE assessment. [file PPUL-60-0-s003.docx]

# Supplement 2

## Table S1

Table 1: Cumulative risk of bias for individual studies

| **Author & year** | **Risk of bias** |
| --- | --- |
| *Akinbami et al. (2014)* | High risk of bias |
| *Grunwell et al. (2018)* | Some concerns |
| *Lee et al. (2023)* | Some concerns |
| *Radhakrishnan et al. (2018)* | Low risk of bias, apart from concerns related to residual confounding |
| *van den Bosch et al. (2012)* | Very high risk of bias |
| *Simms-Williams et al. (2024)* | Some concerns |

## Table S2

Supplementary Table 2: Risk of bias for individual studies across 7 domains

| **Author & year** | Domain 1 | Domain 2 | Domain 3 | Domain 4 | Domain 5 | Domain 6 | Domain 7 |
| --- | --- | --- | --- | --- | --- | --- | --- |
| *Akinbami et al. (2014)* | High risk | Low risk | Low risk | Low risk | Some concern | Low risk | Low risk |
| *Grunwell et al. (2018)* | Low risk | Low risk | Some concern | Low risk | Low risk | Low risk | Low risk |
| *Lee et al. (2023)* | Low risk | Low risk | Some concern | Low risk | Some concern | Low risk | Low risk |
| *Radhakrishnan et al. (2018)* | Low risk | Low risk | Low risk | Low risk | Low risk | Low risk | Low risk |
| *van den Bosch et al. (2012)* | *Very high risk of bias** | | | | | | |
| *Simms-Williams et al. (2024)* | Low risk | Low risk | Some concern | Low risk | Some concern | Low risk | Low risk |

Risk of bias domains: 1) risk of bias due to confounding, 2) risk of bias arising from the measurement of exposure 3) risk of bias due to selection of the participants into the study, 4) risk of bias due to post-exposure interventions, 5) risk of bias due to missing data, 6) risk of bias arising from the measurement of outcomes, 7) risk of bias in the selection of reported result
*note that the risk of bias in the Van den Bosch et al. (2012) study was not assessed across individual domains as per the ROBINS-E preliminary assessment the study was deemed as having a very high risk of bias and no further detailed assessment was recommended.

## Table S3

Supplementary Table 3: GRADE assessment for the outcome: mortality

| GRADE domain | Judgement | Concerns about certainty domains |
| --- | --- | --- |
| Methodological limitations of the studies | Only one study reported on mortality as an outcome and had a high risk of bias due to not adjusting for confounding. | Serious |
| Indirectness | The study directly addressed the clinical question | Not serious |
| Imprecision | Neither the number of events nor the incidence rate were reported for the full analysed period. | Serious |
| Inconsistency | Unable to assess as no comparison with other studies. | Not suspected |
| Publication bias | There was a comprehensive search performed for the studies and the risk of publication bias is low. | Not suspected |

## Table S4

Supplementary table 4: GRADE assessment for the outcome: ICU/HDU admission

| GRADE domain | Judgement | Concerns about certainty domains |
| --- | --- | --- |
| Methodological limitations of the studies | Studies were assessed for risk of bias across 7 domains: 1) risk of bias due to confounding, 2) risk of bias arising from the measurement of exposure 3) risk of bias due to selection of the participants into the study, 4) risk of bias due to post-exposure interventions, 5) risk of bias due to missing data, 6) risk of bias arising from the measurement of outcomes, 7) risk of bias in the selection of reported result. One study ^20^ was deemed to have a low risk of bias across all of the domains, this study had a large number of participants but neither the relative risk estimate nor the number of events for the ICU/HDU admission as an outcome were provided. One study ^21^ was assessed as having a very high risk of bias due to not adjusting for confounders, this study was a case-control study with a very small number of participants in the case group (n=66). Three other studies ^18; 19; 22^ raised some concerns related to selection bias. Two studies ^19; 22^ had a risk of bias due to missing data. | Serious |
| Indirectness | The choice of patients, exposures and comparators across the study directly answers the research question. The outcome measure is clear across the studies, however, the criteria for ICU admission may vary between the countries where the studies were conducted. | Not serious |
| Imprecision | The total number of participants in all of the included observational studies was nearly 2mln (1 998 755). One case-control study had only 230 participants, of which 66 were the cases ^21^. As ICU admission is a binary outcome, the number of events was analysed across the studies from 66 ^21^ to 170 ^18^. In the Simms-Williams et al. (2024) study there were 92 PICU admissions in the 12-17 age group and 137 in the 5-11 age group, while Lee et al. (2023) reported 107 admissions. Neither the number of events nor the incidence rate for ICU/HDU admission was provided for the Radhakrishnan et al. (2018) study. The studies used different measurement units (odds ratio, incidence rate ratio, hazard ratio) but the confidence intervals in all studies for the reported risk factors were wide indicating a level of imprecision. | Not serious |
| Inconsistency | Statistical measures of heterogeneity cannot be applied to the studies included. Different studies reported on different risk factors influencing the outcome. There is a visible trend for specific risk factors to be considered high risk for ICU admission across the studies (e.g. Black ethnicity, previous hospitalisation, allergies, low socioeconomic status, being a female). Remaining risk factors, such as specific medication use (SABA, ICS, OCS, LTRA), other comorbidities (e.g. anxiety, eczema, GORD, allergic rhinitis etc.), family history, smoking exposure etc. were only reported in single studies, hence assessment of inconsistency is not possible. | Not serious |
| Publication bias | There was a comprehensive search performed for the studies and the risk of publication bias is low. | Not suspected |
