# Supplementary material for: Risk Factors for Life‐Threatening Asthma Attacks and Asthma‐Related Mortality in Children—A Systematic Review
Source: Pediatr Pulmonol. 2025 Aug 19;60(8):e71255. doi: 10.1002/ppul.71255 (PMC12391745; doi:10.1002/ppul.71255)
Supplement: Supplementary file 3 — Supplement 3 ‐ Risk factors for ICU admission‐tabular presentation. [file PPUL-60-0-s002.docx]

## Supplement 3

Risk factors for ICU admission grouped by themes

| Risk factor | Study name | Specific Variable | Outcome measure | 95% CI | p-value |
| --- | --- | --- | --- | --- | --- |
| Hospitalisation | **Grunwell** | Hospitalised in last 12 months | OR 8.19 | 4.83 - 13.89 | <0.001 |
|  | **Van den Bosch** | Previous non-PICU hospitalisation | OR 5.4 | 1.34 - 21.45 | 0.02 |
| Low socioeconomic background | **Grunwell** | %below poverty line | OR 1.28 | 1.02 - 1.61 | 0.037 |
|  | **Lee** | low socioeconomic status- on medical aids | aHR 7.12 | 3.72 - 13.62 |  |
|  | **Radhakrishnan** | instability quintile (1 unit increase reference) | OR 1.09 | 0.99 - 1.19 | 0.07 |
|  |  | dependency quintile (1 unit increase reference) | OR 0.95 | 0.87 - 1.04 | 0.3 |
|  |  | ethnic concentration quintile (1 unit increase reference) | OR 0.90 | 0.82 - 0.99 | 0.03 |
|  |  | deprivation quintile (1 unit increase reference) | OR 1.16 | 1.07 - 1.27 | <0.005 |
|  | **Simms-Williams**  Ages 5-11 | IMD 1 (ref) | IRR 1 |  |  |
|  |  | IMD 2 | IRR 1.41 | 0.68 - 2.93 | 0.358 |
|  |  | IMD 3 | IRR 1.59 | 0.78 - 3.22 | 0.202 |
|  |  | IMD 4 | IRR 1.63 | 0.82 - 3.22 | 0.16 |
|  |  | IMD 5 | IRR 1.98 | 1.03 - 3.79 | 0.041 |
|  | **Simms-Williams**  Ages 12-17 | IMD 1 (ref) | IRR 1 |  |  |
|  |  | IMD 2 | IRR 2.08 | 0.83 - 5.19 | 0.117 |
|  |  | IMD 3 | IRR 2.32 | 0.95 - 5.66 | 0.066 |
|  |  | IMD 4 | IRR 2.42 | 1.01 - 5.77 | 0.046 |
|  |  | IMD 5 | IRR 2.09 | 0.88 - 4.98 | 0.096 |
| Allergies | **van den Bosch** | Allergies | OR 5.2 | 1.14 - 23.42 | 0.03 |
|  | **Simms-Williams** | Allergies 5-11 | IRR 1.16 | 0.74 - 1.83 | 0.52 |
|  | **Simms-Williams** | Allergies 12-17 | IRR 1.51 | 0.89 - 2.54 | 0.124 |
| Ethnicity | **Grunwell** | Black ethnicity (white ref) | OR 2.01 | 1.05 - 3.84 | 0.034 |
|  |  | Other | OR 1.3 | 0.53 - 3.2 | 0.568 |
|  | **Simms-Williams**  Age 5-11 | Black ethnicity (white ref) | IRR 4.07 | 2.35 - 7.05 | <0.001 |
|  |  | Mixed | IRR 2.49 | 1.23 - 5.05 | 0.011 |
|  |  | Asian | IRR 1.17 | 0.65 - 2.12 | 0.598 |
|  |  | Other | IRR 1.26 | 3 - 5.35 | 0.75 |
|  | **Simms-Williams**  Age 12-17 | Black ethnicity (white ref) | IRR 3.51 | 1.62 - 7.59 | 0.001 |
|  |  | Mixed | IRR 2.43 | 0.9 - 6.54 | 0.08 |
|  |  | Asian | IRR 1.46 | 0.73 - 2.93 | 0.287 |
|  |  | Other | No data |  |  |
| Sex | **Radhakrishnan** | Male vs. female (ref) | OR 0.93 | 0.76 - 1.15 | 0.5 |
|  | **Simms-Williams, 5-11** | Female vs. male (ref) | IRR 1.20 | 0.85 - 1.7 | 0.306 |
|  | **Simms-Williams, 12-17** | Female vs. male (ref) | IRR 1.54 | 1 - 2.36 | 0.048 |
| Age | **Grunwell** | Age >12 | 2.31 | 1.39 - 3.86 | 0.001 |
|  | **Radhakrishnan** | Age at diagnosis (1 unit increase) | 0.92 | 0.87 - 0.97 | <0.005 |
| Comorbidities | **Grunwell** | **Pneumonia** | OR 2.56 | 1.52 - 4.29 | <0.001 |
|  | **Radhakrishnan** | **Comorbidity (1 vs 0)** | OR 1.87 | 1.44 - 2.42 | p <0.005 |
|  | **Simms-Williams**  **5-11** | BMI (normal weight ref)  Underweight  Overweight  Obese  Missing | IRR 1.08  IRR 1.53  IRR 0.67  IRR 1.47 | 0.47 – 2.45  0.85 – 2.74  0.23 – 1.9  0.97 - 2.21 | 0.858  0.158  0.448  0.067 |
|  |  | Atopic eczema | IRR 1.02 | 0.72 - 1.46 | 0.894 |
|  |  | Allergic rhinitis | IRR 0.67 | 0.40 - 1.14 | 0.141 |
|  |  | GORD | IRR 0.57 | 0.23 - 1.43 | 0.233 |
|  |  | Chronic rhinosinusitis | No data |  |  |
|  |  | Anxiety | IRR 0.76 | 0.10 - 5.75 | 0.791 |
|  |  | Depression | IRR 0.79 | 0.10 - 6.11 | 0.825 |
|  | **Simms-Williams 12-17** | BMI (normal weight ref)  Underweight  Overweight  Obese  Missing | IRR 1.04  IRR 0.91  IRR 0.85  IRR 0.86 | 0.53 – 2.06  0.48 – 1.73  0.38 – 1.91  0.48 - 1.54 | 0.900  0.781  0.701  0.618 |
|  |  | Atopic eczema | IRR 0.86 | 0.56 - 1.35 | 0.521 |
|  |  | Allergic rhinitis | IRR 1.05 | 0.62 - 1.77 | 0.854 |
|  |  | GORD | IRR 1.14 | 0.40 - 3.27 | 0.806 |
|  |  | Chronic rhinosinusitis | No data |  |  |
|  |  | Anxiety | IRR 0.94 | 0.36 - 2.46 | 0.906 |
|  |  | Depression | IRR 1.90 | 0.77 - 4.70 | 0.166 |
| Medication | **Grunwell** | **Severe asthma/high ICS** | OR 2.76 | 1.62 - 4.70 | 0.001 |
|  | **Simms-Williams**  **5-11** | **SABA prescriptions per year (0 ref)**  1-3 **4-6  7+** | IRR 2.13  IRR 5.21  IRR 6.97 | 0.98 – 4.61  2.26 – 12.01  2.91 – 16.68 | 0.056  p<0.001  p<0.001 |
|  |  | **OCS** | IRR 1.98 | 1.34 - 2.92 | 0.001 |
|  |  | ICS | IRR 1.31 | 0.73 - 2.34 | 0.371 |
|  |  | LABA | IRR 1.23 | 0.43 - 3.53 | 0.699 |
|  |  | **LTRA** | IRR 2.86 | 1.93 - 4.24 | p <0.001 |
|  |  | Influenza vaccine | IRR 1.00 | 0.70 - 1.44 | 0.988 |
|  | **Simms-Williams 12-17** | **SABA prescriptions per year (0 reference)**  1-3  4-6 **7+** | IRR 1.71  IRR 3.03 IRR 8.44 | 0.53 – 5.50  0.86 – 10.64  2.49 – 28.56 | 0.369  0.083  0.001 |
|  |  | **OCS** | IRR 4.06 | 2.56 - 6.45 | p <0.001 |
|  |  | **ICS** | IRR 3.95 | 1.40 - 11.17 | 0.009 |
|  |  | LABA | IRR 1.72 | 0.78 - 3.79 | 0.180 |
|  |  | **LTRA** | IRR 2.77 | 1.71 - 4.49 | p <0.001 |
|  |  | Influenza vaccine | IRR 0.89 | 0.57 - 1.39 | 0.616 |
| Environmental factors | **Radhakrishnan** | **Rural vs urban (ref)** | OR 2.42 | 1.80 - 3.25 | p <0.005 |
|  | **Simms-Williams 12-17** | Smoking (never smoked ref)  Current smoker  Former smoker  Missing | IRR 0.83  IRR 1.14 IRR 1.09 | 0.40 – 1.73  0.65 – 2.01  0.55 – 2.13 | 0.616  0.655  0.807 |
| Other factors | **Radhakrishnan** | **Year of birth (1 unit increase)** | OR 1.17 | 1.12 - 1.21 | p <0.005 |
|  |  | **Paediatrician vs non-=paediatrician** | OR 0.74 | 0.58 - 0.94 | p= 0.010 |
|  | **Grunwell** | **Father with asthma** | OR 2.15 | 1.23 - 3.76 | p=0.007 |
